# In Silico Analysis of PKS and NRPS Gene Clusters in Arisostatin- and Kosinostatin-Producers and Description of *Micromonospora okii* sp. nov.

**DOI:** 10.3390/antibiotics10121447

**Published:** 2021-11-25

**Authors:** Hisayuki Komaki, Natsuko Ichikawa, Akira Hosoyama, Moriyuki Hamada, Yasuhiro Igarashi

**Affiliations:** 1 Biological Resource Center, National Institute of Technology and Evaluation (NBRC), Chiba 292-0818, Japan; hamada-moriyuki@nite.go.jp; 2 Biological Resource Center, National Institute of Technology and Evaluation (NBRC), Tokyo 151-0066, Japan; ichikawa-natsuko@nite.go.jp (N.I.); hosoyama-akira@nite.go.jp (A.H.); 3 Biotechnology Research Center and Department of Biotechnology, Toyama Prefectural University, Toyama 939-0398, Japan; yas@pu-toyama.ac.jp

**Keywords:** arisostatin, classification, kosinostatin, *Micromonospora*, polyketide, quinolidomicin, secondary metabolite

## Abstract

*Micromonospora* sp. TP-A0316 and *Micromonospora* sp. TP-A0468 are producers of arisostatin and kosinostatin, respectively. *Micromonospora* sp. TP-A0316 showed a 16S rRNA gene sequence similarity of 100% to *Micromonospora* *oryzae* CP2R9-1^T^ whereas *Micromonospora* sp. TP-A0468 showed a 99.3% similarity to *Micromonospora haikouensis* 232617^T^. A phylogenetic analysis based on *gyrB* sequences suggested that *Micromonospora* sp. TP-A0316 is closely related to *Micromonospora oryzae* whereas *Micromonospora* TP-A0468 is an independent genomospecies. As *Micromonospora* sp. TP-A0468 showed some phenotypic differences to its closely related species, it was classified as a novel species, for which the name *Micromonospora okii* sp. nov. is proposed. The type strain is TP-A0468^T^ (= NBRC 110461^T^). *Micromonospora* sp. TP-A0316 and *M. okii* TP-A0468^T^ were both found to harbor 15 gene clusters for secondary metabolites such as polyketides and nonribosomal peptides in their genomes. Arisostatin-biosynthetic gene cluster (BGC) of *Micromonospora* sp. TP-A0316 closely resembled tetrocarcin A-BGC of *Micromonospora chalcea* NRRL 11289. A large type-I polyketide synthase gene cluster was present in each genome of *Micromonospora* sp. TP-A0316 and *M. okii* TP-A0468^T^. It was an ortholog of quinolidomicin-BGC of *M. chalcea* AK-AN57 and widely distributed in the genus *Micromonospora*.

## 1. Introduction

**Figure 1:**
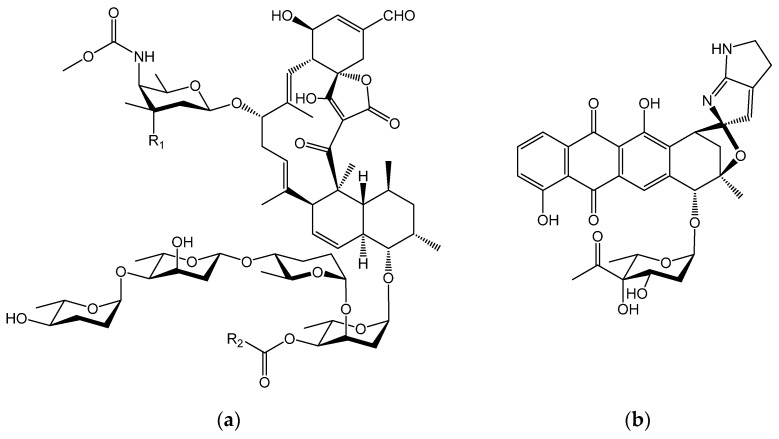
Chemical structures of arisostatins A and B and tetrocarcin A (**a**) and kosinostatin (**b**). Arisostatin A, R_1_ = NO_2_, R_2_ = CH(CH_3_)_2_; arisostatin B, R_1_ = NH_2_, R_2_ = CH(CH_3_)_2_; tetrocarcin A: R_1_ = NO_2_, R_2_ = CH_3_.

Actinomycetes are Gram-positive filamentous bacteria and its members are recognized as a rich source of bioactive secondary metabolites, many of which have been utilized for pharmaceutical purposes [1]. Although soil is the main habitat of actinomycetes, including the genus *Streptomyces*, marine environments such as sea water have been identified as sites for the isolation of actinomycetal strains producing new bioactive compounds. Members of the genus *Micromonospora* are often isolated from marine environments and have been found to produce diverse secondary metabolites [2]. In our previous studies, *Micromonospora* sp. TP-A0316 and *Micromonospora* sp. TP-A0468 were isolated from sea water by the membrane filter method, followed by their cultivation on an agar plate [3,4]. *Micromonospora* sp. TP-A0316 produces novel compounds, named arisostatins A and B, in addition to tetrocarcin A [3] whereas *Micromonospora* sp. TP-A0468 produces kosinostatin [4]. Arisostatins are new members of the tetrocarcin class of antibiotics (Figure 1a), providing antibiotic activity against Gram-positive bacteria and demonstrating antitumor activity [3]. Although the tetrocarcin A-biosynthetic gene cluster (BGC) was identified in *Micromonospora chalcea* NRRL 11289 [5], the arisostatin-BGC of *Micromonoposra* sp. TP-A0316 has not yet been identified. Kosinostatin is a new quinocycline antibiotic (Figure 1b) with antibacterial, anti-yeast and antitumor activities [4]. Kosinostatin-BGC have already been reported in *Micromonospora* sp. TP-A0468. Tetrocarcin A and kosinostatin are synthesized via type-I polyketide synthase (PKS) and type-II PKS pathways, respectively [5,6].

Polyketides are biosynthesized by the assembly of acyl-CoA units. Type-I PKSs are large modular enzymes composed of multiple catalytic domains and synthesize polyketide chains based on the co-linearity rule of assembly lines. The mechanism resembles that of nonribosomal peptide synthetase (NRPS) pathways, as nonribosomal peptides are biosynthesized by the assembly of amino acid units and NRPSs are also large modular enzymes composed of multiple catalytic domains and synthesize peptide chains according to the co-linearity rule of assembly lines [7]. In type-II PKS pathways, a set of three enzymes, ketosynthase α (KSα), KSβ (chain length factor), and acyl carrier protein (ACP), iteratively catalyzes the elongation of polyketide chains. The products are mainly aromatic compounds [8]. Approximately half to three quarters of secondary metabolite-BGCs, in the genomes of actinomycetes, are associated with PKS or NRPS pathways. This suggests that polyketides, nonribosomal peptides, and their hybrid compounds, which are synthesized by hybrid PKS/NRPS gene clusters, are major secondary metabolites in actinomycetes [9]. These compounds are structurally diverse and often exhibit useful pharmaceutical activities. Hence, nowadays, genome analyses focusing on PKS and NRPS gene clusters are often conducted to evaluate the potential use of actinomycete strains as a source for novel secondary metabolites [10,11,12].

In this study, we investigated the taxonomic positions of *Micromonospora* sp. TP-A0316 and *Micromonospora* sp. TP-A0468, since the classification of antibiotic producers at the species level is important to understand the relationship between species and products. Next, we sequenced whole genomes of these two strains to reveal their potential in producing diverse secondary metabolites such as polyketides and nonribosomal peptides. Consequently, *Micromonospora* sp. TP-A0468 was considered to be a novel species, for which we propose *Micromonospora okii* sp. nov. Additionally, we observed a wide distribution of quinolidemicin-BGCs in the genus *Micromonospora* and classified ten *Micromonospora* strains for which whole genome sequences have been published, although species names have been unclear.

## 2. Results

### 2.1. Classification of Micromonospora sp. TP-A0316 and Micromonospora sp. TP-A0468

**Figure 2:**
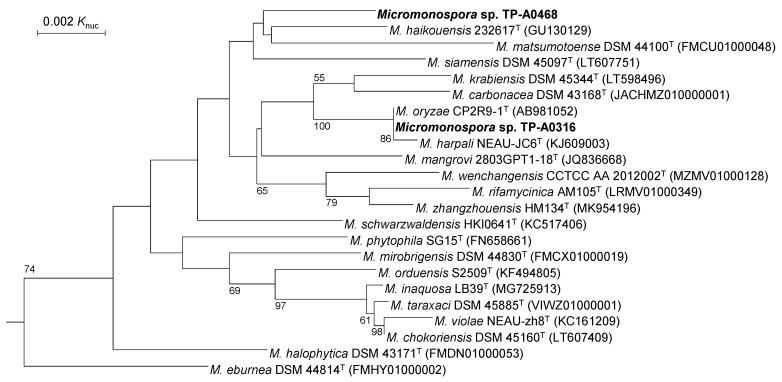
Phylogenetic tree based on 16S rRNA gene sequences. Numbers on the branches represent the confidence limits estimated by bootstrap analysis with 1000 replicates; values above 50% are at branching points. *Phytohabitans suffuscus* K07-0523^T^ (AB490769) was used as an outgroup (not shown).

*Micromonospora* sp. TP-A0316 showed a 16S rRNA gene sequence similarity of 100% to *Micromonospora oryzae* CP2R9-1^T^ whereas *Micromonospora* sp. TP-A0468 showed a similarity of 99.3% to *Micromonospora haikouensis* 232617^T^. In the phylogenetic tree, based on 16S rRNA gene sequences, *Micromonospora* sp. TP-A0316 formed a clade with the *M. oryzae* and *Micromonospora harpali* strains. In contrast, *Micromonospora* sp. TP-A0468 did not form a clade with any strains with a bootstrap value of >50% (Figure 2).

**Figure 3:**
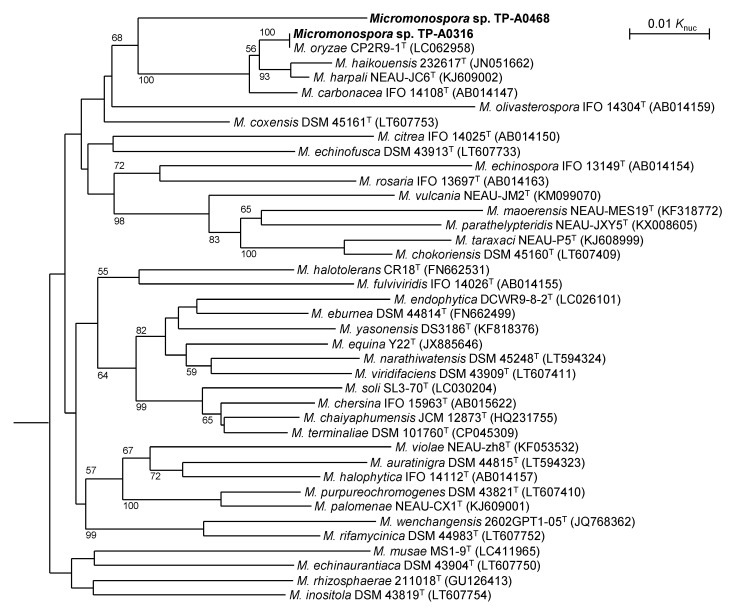
Phylogenetic tree based on *gyrB* sequences. Numbers on the branches represent the confidence limits estimated by bootstrap analysis with 1000 replicates; values above 50% are at branching points. *P. suffuscus* NBRC 105367^T^ (AP022871) was used as an outgroup (not shown).

Next, we reconstructed a phylogenetic tree based on DNA gyrase subunit B gene (*gyrB*) sequences (Figure 3), because 16S rRNA gene-based phylogenies of the genus *Micromonospora* did not always agree with other taxonomic characteristics, and the *gyrB* sequence has been reported to be suitable for phylogenetic classification and identification [13]. *Micromonospora* sp. TP-A0316 formed a clade with the type strain of *M. oryzae* and their *gyrB* sequences are identical. This suggests that *Micromonospora* sp. TP-A0316 is likely *M. oryzae*. On the other hand, the position of *Micromonospora* sp. TP-A0468 was deep branched and monophelic, suggesting its phylogenetical independency. Although *Micromonospora* sp. TP-A0468 formed a clade with the type strains of *M. oryzae, Micromonospora carbonacea, M. harpali* and *M.* *haikouensis*, its *gyrB* sequence similarities to the four strains were 94.9%, 94.9%, 94.9% and 94.7%, respectively. It has been reported that a 98.5% *gyrB*-sequence similarity corresponds to 70% DNA–DNA relatedness [13,14]. As the *gyrB* sequence similarities are well below 98.5%, *Micromonospora* sp. TP-A0468 is considered as an independent genomospecies.

**Figure 4:**
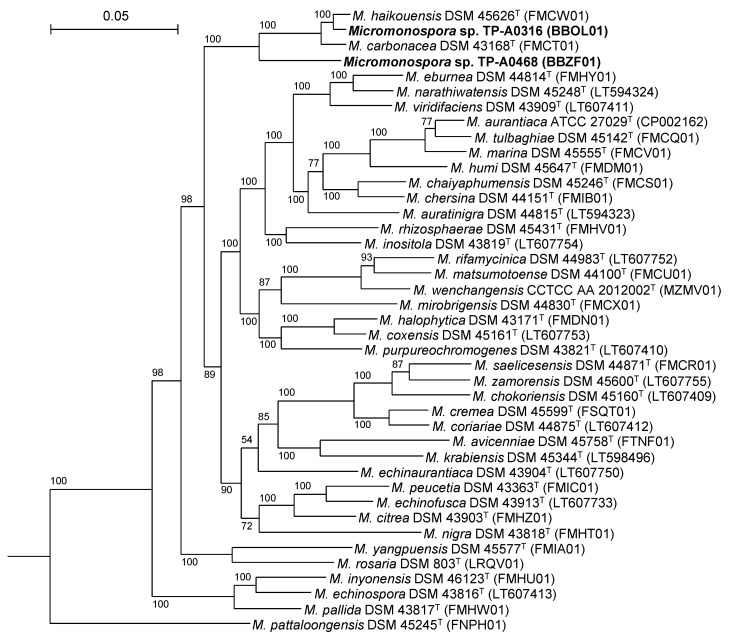
Phylogenetic tree based on MLSA. *Actinoplanes missouriensis* 431^T^ was used as an outgroup (not shown). The numbers in parentheses are accession numbers of whole genome sequences or WGS Projects in GenBank, from which housekeeping gene sequences were obtained.

Additionally, we conducted a multilocus sequence analysis (MLSA) using 85 housekeeping genes (Figure 4). Although *Micromonospora* sp. TP-A0468 formed a clade with *M. haikouensis* DSM 45626^T^, *Micromonospora* sp. TP-A0316 and *M. carbonacea* DSM 43168^T^, its evolutionally relationships with them are not as close as the relationships that exist among the three strains (Figure 4). The DNA–DNA relatedness between *Micromonospora* sp. TP-A0468 and these three members was found to be between 33.5% and 33.8% (data not shown). These results also suggest *Micromonospora* sp. TP-A0468 to be an independent genomospecies.

Phenotypic differences were observed between *Micromonospora* TP-A0468 and its closely related phylogenetic neighbors such as *M. oryzae, M. carbonacea, M. harpali* and *M.* *haikouensis* as listed in Table 1. Unlike these neighbors, *Micromonospora* TP-A0468 includes galactose within the whole-cell sugar. Its growth ranges and utilization pattern of carbon sources are different from those of the other listed species. Although *M. oryzae* may appear to show a similar utilization pattern of carbon sources, except for d-xylose, it produces soluble pigment and liquefies gelatin, which is different to *Micromonospora* TP-A0468. Thus, we classified *Micromonospora* TP-A0468 as a novel species, for which the name *Micromonospora okii* sp. nov. is proposed. The type strain is TP-A0468^T^ (=NBRC 110461^T^).

### 2.2. PKS and NRPS Gene Clusters in Micromonospora sp. TP-A0316 and M. okii TP-A0468^T^

**Figure 5:**
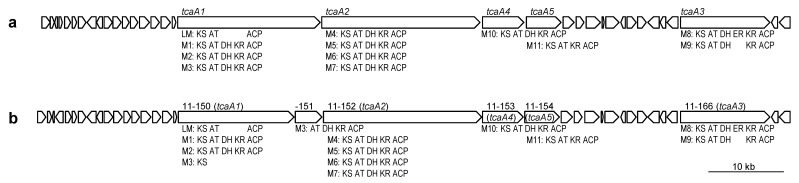
Tetrocarcin A-biosynthetic (*tca*) gene cluster of *M. chalcea* NRRL 11289 (**a**) and *t1pks-1* gene cluster of *Micromonospora* sp. TP-A0316 (**b**); ACP, acyl carrier protein; AT, acyltransferase; DH, dehydratase; ER, enoyl reductase; KR, ketoreductase; KS, ketosynthase; LM, loading module; M, module. Domain organizations are shown below ORFs.

Fifteen gene clusters for secondary metabolites such as polyketides and nonribosomal peptides were observed in the genomes of *Micromonospora* sp. TP-A0316, as listed in Table 2. Type-I PKS gene cluster 1 (*t1pks-1*) resembled the *tca* gene cluster responsible for tetrocarcin A synthesis in *M. chalcea* NRRL 11289 [5] (Figure 5). As arisostatins are congeners of tetrocarcin A, and *Micromonospora* sp. TP-A0316 is reported to produce tetrocarcin A in addition to arisostatins A and B [3], *t1pks-1* was considered as the BGC for arisostatins and tetrocarcin A. Furthermore, *t1pks-2* was found to be a large cluster of >200 kb and include 33 modules. This was considered as an ortholog of BGC for quinolidomicin (*qnm*), the largest known macrolide [18], according to the similar gene and domain organizations (Table 3). However, its module number is different from that of *qnmA* because *t1pks-2* lacks module 4. The product is likely a quinolidomicin congener, but its polyketide skeleton is presumed to be different from quinolidomicin A_1_ [18]. In contrast, *t1pks-3* was not found to be multimodular, but harbored only a single module. This gene cluster was predicted to be involved in sporolide synthesis [19]. As *t1pks-4* was not completely sequenced, its product could not be predicted. Products of *t2pks-1* were not predicted by our bioinformatic analysis. However, it is generally known that type-II PKS pathways are responsible for the synthesis of aromatic compounds. Additionally, *t3pks-1* showed similarity to *agq,* a type-III PKS gene cluster for alkyl-O-dihydrogeranyl-methoxyhydroquinone [20]. Five NRPS gene clusters in this strain did not show high similarities to other known NRPS gene clusters, suggesting them to be orphan, although *nrps-4* was not completely sequenced. They were predicted to synthesize pentapeptide, tripeptide, tetrapeptide and dipeptide, respectively, as listed in Table 2. Four hybrid PKS/NRPS gene clusters, *pks/nrps-1, -2, -3* and *-4*, were also orphan and were predicted to synthesize heptapeptide, tripeptide and pentapeptide with polyketide moieties and hexaketide with a glycine molecule, respectively.

*M. okii* TP-A0468^T^ harbored 6 PKS, 5 NRPS and 4 hybrid PKS/NRPS gene clusters in its genome as shown in Table 4. Moreover, *t1pks-2, t3pks-1, pks/nrps-2* and *pks/nrps-3*, which are asterisked in the tables, were found to be orthologs of gene clusters present in *Micromonospora* sp. TP-A0316. Both *t1pks-5* and *t2pks-2* were responsible for syntheses of 16-demethylrifamycins and kosinostatin, respectively, as reported [6,21]. Although *nrps-6* was not completely sequenced, it is predicted to be a pyochelin-BGC since the homologs are often annotated as pyochelin synthetases. Although *pks/nrps-5* resembled tallysomycin-BGC, ORF 21-45 contained a methyltransferase (MT) domain that is not encoded in *tlmVIII*. As the other domain organization showed good agreement with that of *tlm* gene cluster [22], its product was presumed to be methyltallysomycin. The other gene clusters such as *t2pks-3, t3pks-2, nrps-7* to *-10*, and *pks/nrps-6* were orphan and their products were predicted as shown in Table 4.

### 2.3. Distribution of Quinmuinolidomicin-BGC Orthologs in the Genus Micromonospora

**Figure 6:**
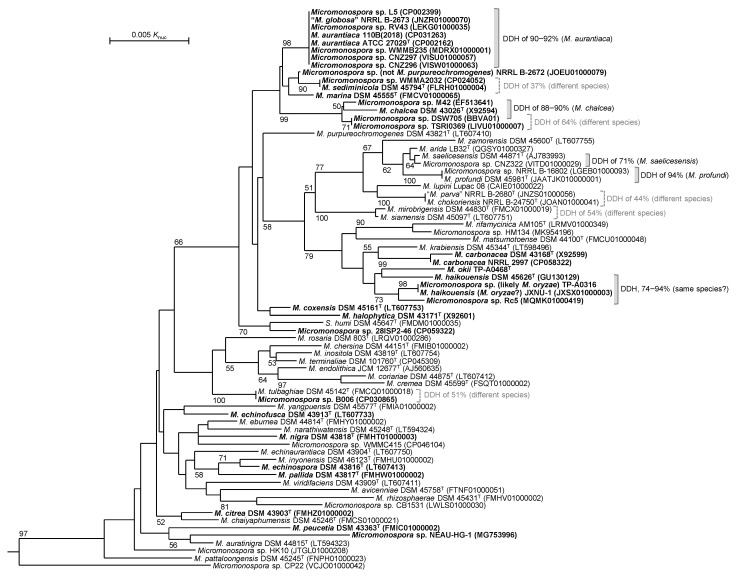
Distribution of quinolidomicin-biosynthetic gene cluster orthologs in the genus *Micromonospora*. Whole genome sequence-published strains are shown in a phylogenetic tree based on 16S rRNA gene sequences. Accession numbers of 16S rRNA gene sequences are shown in parentheses. Strains harboring the ortholog are boldfaced. DDH, DNA-DNA relatedness values determined by digital DNA-DNA hybridization. *P.* *suffuscus* K07-0523^T^ (AB490769) is used as an outgroup (not shown).

Unexpectedly, both *Micromonospora* sp. TP-A0316 and *M. okii* TP-A0468^T^ possessed an ortholog of *qnm* gene cluster, which is the largest type-I PKS gene cluster identified to date [18]. We investigated its distribution in genome sequence-published strains of the genus *Micromonospora*. Among the 74 strains shown in Figure 6, 34 strains were found to harbor the ortholog. Among them, 23 strains were phylogenetically close to *Micromonospora aurantiaca* or to the two strains studied here. However, the remaining 11 strains are phylogenetically diverse, suggesting the ortholog is widely distributed in the genus *Micromonospora*.

Although the 16S rRNA gene sequences between *Micromonospora* sp. B006 and *Micromonospora tulbaghiae* DSM 45142^T^ were identical, it was found that *Micromonospora* sp. B006 harbors the ortholog [23] while *M. tulbaghiae* DSM 45142^T^ does not. Because it is reported that members in the same species possess similar sets of PKS and NRPS gene clusters [24], we examined DNA-DNA relatedness values, which were estimated using digital DNA-DNA hybridization (DDH) among strains showing high 16S rRNA gene sequence similarities to clarify their taxonomic relationships. As noted in Figure 5, the DNA-DNA relatedness value between *Micromonospora* sp. B006 and *M. tulbaghiae* DSM 45142^T^ was 51%, which is below the species cut-off value of 70% defined in the bacteria systematics [25], suggesting them to be different species. *Micromonospora* sp. L5, *Micromonospora* sp. RV43, *Micromonospora* sp. WMMB235, *Micromonospora* sp. CNZ297, *Micromonospora* sp. CNZ296 and “*Micromonospora globosa*” NRRL B-2673 showed a DNA-DNA relatedness value of >90% to the type strain of *M. aurantiaca*. *Micromonospora* sp. M42 and *Micromonospora* sp. DSW705 showed ~90% to the type strain of *M. chalcea*, but *Micromonospora* sp. TSRI0369 did not. In contrast, the DNA-DNA relatedness values between *Micromonospora* sp. WMMA2032 and *Micromonospora sediminicola* DSM 45794^T^, *Micromonospora* sp. DSW705 and *Micromonospora* sp. TSRI0369, and *M. parva* NRRL B-2680^T^ and *M. chokoriensis* NRRL B-24750^T^ were less than 70% although these strain pairs shared the same 16S rRNA gene sequence. The DNA-DNA relatedness values between *M. saelicesensis* DSM 44871^T^ and *Micromonospora* sp. CNZ322, *Micromonospora* sp. NRRL B-16802 and *M. profundi* DSM 45981^T^, and among *Micromonospora* sp. TP-A0316, *M. haikouensis* JXNU-1, *M. haikouensis* DSM 45626^T^ and *Micromonospora* sp. Rc5 were 71%, 94% and 74–94%, respectively.

## 3. Discussion

The relationships that exist between taxonomic species and secondary metabolites are still unclear because many strains that produce bioactive secondary metabolites have not been classified at species level. This study aimed to elucidate the taxonomic positions of both *Micromonospora* sp. TP-A0316, a producer of arisostatins, and *Micromonospora* sp. TP-A0468, a producer of kosinostatin, at the species level. We concluded that *Micromonospora* sp. TP-A0316 is closely related to *M. oryzae,* and that *Micromonospora* sp. TP-A0468 should be classified as a novel species, for which we propose *M. okii* sp. nov. These two strains each harbor 15 PKS and NRPS gene clusters in their genomes. We characterized these gene clusters bioinformatically. Among the 15 clusters of each strain, only 4 were conserved between the strains. This is because *Micromonospora* sp. TP-A0316 and *M. okii* TP-A0468^T^ are different species.

Our genome analysis revealed that, alongside the two strains that have not been reported as quinolidomicin-producers, diverse *Micromonospora* strains harbor orthologs of the *qnm* gene cluster. Members in the genus *Micromonospora* are known to include producers of aminoglycoside antibiotics such as gentamicin [26], mutamicin [27], netilmicin, retymicin, sisomicin [28], verdamicin and turbinmicin [29]. Quinolidomicins may be one of the representative products, although the report is limited [30] by the difficulties associated with its structure [18,31].

In addition to *Micromonospora* sp. TP-A0316 and *Micromonospora* sp. TP-A0468, many genome sequence-published *Micromonospora* strains have not been classified at species level. Digital DDH conducted in this study clarified the taxonomic positions as follows: *Micromonospora* sp. L5, *Micromonospora* sp. RV43, *Micromonospora* sp. WMMB235, *Micromonospora* sp. CNZ297, *Micromonospora* sp. CNZ296 and *M. globosa* NRRL B-2673 are *M. aurantiaca*; *Micromonospora* sp. M42 and *Micromonospora* sp. DSW705 are *M. chalcea*; *Micromonospora* sp. NRRL B-16802 is *M. profundi*. Although the strain NRRL B-2672 has been published as *Micromonospora purpureochromogenes*, we found this to not be true, because they are phylogenetically distant as shown in Figure 5 and its DNA-DNA relatedness to *M. purpureochromogenes* DSM 43827^T^ was only 27% (data not shown). It may be possible to classify *Micromonospora* sp. CNZ322 as *M. saelicesensis* since their DNA-DNA relatedness was found to be 71%. In contrast, *Micromonospora* sp. WMMA2032, *Micromonospora* sp. TSRI0369 and *Micromonospora* sp. B006 are likely to be classified as independent genomospecies, since their DNA-DNA relatedness to each phylogenetic neighbor was 37%, 64% and 51%, respectively.

We stated that *Micromonospora* sp. TP-A0316 is likely to be classified as *M. oryzae* in the results section. However, this strain and *M. haikouensis* JXNU-1, which is not the type strain of *M. haikouensis,* unexpectedly shared the same 16S rRNA gene sequence as shown in Figure 6. Strain JXNU-1 may not be *M. haikouensis* but *M. oryzae.* Our digital DNA–DNA hybridization suggested that *M. oryzae* and *M. haikouensis* may be identical because the members showed DNA–DNA relatedness values of >74%, as shown in Figure 5, although whole genome sequence of *M. oryzae* type strain is not available. If it is considered that *M. oryzae* and *M. haikouensis* are synonym, *Micromonospora* sp. TP-A0316 may be classified as *M. haikouensis* based on the priority rule of the International Code of Nomenclature of Bacteria.

## 4. Description of *Micromonospora okii* sp. nov.

*Micromonospora okii* (o.ki’i. N.L. gen. n. *okii* of Oki, named in honor of the late Professor Toshikazu Oki, a celebrated actinomycete biologist who organized the study on strain TP-A0468).

The description provided is based on data obtained in a previous study [4]. Aerobic and Gram stain-positive filamentous actinomycete. Spores are singly formed on substrate mycelium. The spore shape and size are oval and range from 0.8 to 1.2 mm, respectively. The colors of vegetative mycelium and the reverse side are yellowish or grayish white to grayish brown on sucrose-nitrate agar, white light orange on glucose-asparagine agar, yellowish brown on Bennett’s agar, light orange to dark gray on nutrient agar, light or light yellowish brown to grayish brown on oatmeal agar, dark brown to dark yellowish brown on inorganic salts-starch agar, and white on glycerol asparagine agar. Vegetative mycelium and the reverse side are, respectively, beige white to light grayish brown and white on glucose-nitrate agar, soft orange to olive gray and light yellowish brown to medium gray on yeast extract-malt extract agar, beige gray to light yellowish brown and grayish white to yellowish brown on tyrosine agar. Vegetative mycelium acts well on nutrient agar, Bennett’s agar, yeast extract-malt extract agar, oatmeal agar, and inorganic salts-starch agar, but poorly on sucrose-nitrate agar, glucose-nitrate agar, glucose-asparagine agar, glycerol asparagine agar and tyrosine agar. Aerial mycelium and diffusible pigments are not formed. Starch hydrolysis, milk coagulation and milk peptonization are positive. The temperature range for growth is 13 to 41 °C and the optimum temperature is from 25 to 39 °C. d-Glucose, sucrose maltose, l-rhamnose, d-mannose, d-fructose, l-arabinose, and d-galactose are utilized for growth. Inositol, d-mannitol, raffinose and d-xylose are not utilized. Whole-cell hydrolysates contain *meso*-diaminopimelic acid as the diagnostic diamino acid, and galactose, xylose, arabinose and glucose as the whole-cell sugars. The phospholipid type is the PII pattern, and phosphatidylethanolamine and phosphatidylinositol are present. The type strain produces kosinostatin.

The type strain is TP-A0468^T^ (=NBRC 110461^T^). The DNA G+C content of the type strain is 73.9% (determined by whole genome-sequencing). Accession numbers of the draft genome sequence of the type strain are BBZF01000001–BBZF01000036.

## 5. Materials and Methods

*Micromonospora* sp. TP-A0316 and *Micromonospora* sp. TP-A0468 were isolated as previously reported [3,4] and were deposited onto the NBRC culture collection as NBRC 110038 and NBRC 110461, respectively. The 16S rRNA gene sequences were determined by the same method used in our previous report [32]. The EzBioCloud web server [33] was used to search closely related type strains and calculate 16S rRNA gene sequence similarities. Phylogenetic trees based on 16S rRNA gene and *gyrB* sequences were reconstructed by the neighbor-joining method using ClustalX 2.1. MLSA was conducted by autoMLST [34] using the DNA sequences of 85 housekeeping genes: *gatB*, *gatA*, amino acid biosynthesis phosphoglycerate dehydrogenase (PGDH) gene, amino acid biosynthesis acetolactate synthase, small subunit (acolac_sm) gene, imidazole glycerol phosphate synthase, glutamine amidotransferase subunit (IMP_synth_hisH) gene, *nuoF*, phosphoribosylformylglycinamidine synthase II (FGAM_synth_II) gene, *rsmG, typA/bipA, ilvD*, phosphoribosylformylglycinamidine synthase I (FGAM_synth_I) gene, *hutU, yjeE*, fructose-bisphosphate aldolase, class II (FruBisAldo_II_A) gene, histidinol-phosphatase (his_9_HisN) gene, *recQ, nth, whiA*, transketolase (tktlase_bact) gene, polyphosphate kinase 1 (poly_P_kin) gene, *atpD, rplA, hrcA, glpX, rpe, lipA, purH*, translation initiation factor IF-2 gene, *pdx2*, SUF system FeS assembly protein, NifU family (SUF_scaf_2) gene, *ung, rplM, atpA, secA, gyrA*, preprotein translocase, SecY subunit (3a0501s007) gene, *prfA, rpsC, truB, rplS*, cystathionine beta-synthase (cysta_beta) gene, *pth, pyrG*, ribonuclease PH (RNasePH) gene, *clpX*, hypoxanthine phosphoribosyltransferase (HGPRTase) gene, *ftsZ, ftsY, rlmN, cgtA, ftsE, trmU, prfB, radA, rpoC*, CCA tRNA nucleotidyltransferase gene, *ksgA, era*, 1,4-alpha-glucan branching enzyme gene, *ruvB, purS, pyrF, recA, dxs, gyrB, pdx1, engA, ffh, recR, dnaA, sufB, dxr, trmD, rplB, pyrH, mfd, rplV, mraZ, purA*, nicotinate (nicotinamide) nucleotide adenylyltransferase gene, *aspS, rpoZ*, phosphopantothenoylcysteine decarboxylase/phosphopantothenate—cysteine ligase (coaBC_dfp) gene, *purF*, and *rpsB*. Whole genome sequencing and analyses of PKS and NRPS gene clusters in the genome sequences were conducted in the same manner of our previous reports [32,35,36,37]. These gene clusters and their domain were detected and determined, respectively, using antiSMASH [38]. The products were predicted not only through KnownClusterBlast in antiSMASH but also manually, based on module numbers, domain organizations, and substrates of adenylation domains. The draft genome sequences have been published in GenBank/EMBL/DDBJ under the accession numbers of BBOL01000001–BBOL01000026 and BBZF01000001–BBZF01000036, respectively. DNA–DNA relatedness was estimated by digital DDH using Formula 2 of the Genome-to-Genome Distance Calculator 2.1 (GGDC) [39].

## Figures and Tables

**Table 1:** Phenotypic characters different between *Micromonospora* sp. TP-A0468 and closely related species.

Character	1	2	3	4	5
Melanine formation	+	nd	nd	nd	−
Soluble pigment	−	nd	+	nd	−
Whole cell sugar	Gal, Xyl, Ara, Glu	Ara, Xyl, Glu	Ara, Glu, Rib, Xyl	Ara, Glu, Xyl	Glu, Xyl, Man
Phospholipid	PE, PI	PE, DPG, PIM	DPG, PE, PG, PI, PIMs	DPG, PE, PIM	DPG, PE, PIM
Starch hydrolysis	+	+	+	v	+
Milk peptonization	+	nd	+	nd	−
Cellulose decomposition	−	+	nd	+	−
Gelatin liquefaction	+	+	−	nd	−
Utilization of carbon source					
l-Arabinose	+	−	+	−	−
d-Fructose	+	v	+	v	−
d-Galactose	+	v	+	−	+
Inositol	−	nd	−	nd	+
Maltose	+	−	nd	+	+
d-Mannitol	−	v	−	v	+
d-Mannose	+	−	+	+	+
d-Raffinose	−	+	w	+	+
d-Xylose	−	v	+	v	−
Growth temperature (optimum, °C)	13–41 (25–39)	nd	20–45 (30)	nd	15–40 (28)
pH for growth (optimum)	6–10 (7–8)	5–8.5	5–10 (7)	nd	6–10 (7)
NaCl tolerance (%)	<4	3	4	3	3.5

1: *Micromonospora* sp. TP-A0468; 2: *M. haikouensis*; 3: *M. oryzae*; 4: *M. carbonacea*; 5: *M. harpani*; +: positive; −: negative; Ara: arabinose; DPG: diphosphatidylglycerol; Gal: galactose; Glu: glucose; Man: mannose; nd: not determined; PE: phosphatidylethanolamine; PI: phosphatidylinositol; PG: phosphatidylglycerol; PIM: phosphatidylinositol mannoside; Rib, ribose; v: varied; w: weak; Xyl: xylose. These data are taken from previous reports [4,15,16,17].

**Table 2:** PKS and NRPS gene clusters in the genomes of *Micromonospora* sp. TP-A0316.

Cluster	ORF	Domain Organization	Predicted Product
*t1pks-1*	11-150 (*tcaA1*)	KS/AT/ACP-KS/AT/DH/KR/ACP-KS/AT/DH/KR/ACP-KS	arisostatins A & B, tetrocarcin A
	11-151 (*tcaA1*)	AT_mm_/DH/KR/ACP	
	11-152 (*tcaA2*)	KS/AT_m_/DH/KR/ACP-KS/AT/DH/KR/ACP-KS/AT_m_/DH/KR/ACP-	
		KS/AT_m_/DH/KR/ACP	
	11-153 (*tcaA4*)	KS/AT_mm_/DH/KR/ACP	
	11-154 (*tcaA5*)	KS/AT_m_/KR/ACP	
	11-166 (*tcaA3*)	KS/AT/DH/ER/KR/ACP- KS/AT_mm_/DH/KR/ACP	
*t1pks-2* *	1-1073	CoL/KR/ACP-KS/AT_m_/DH/KR/ACP-KS/AT_mm_/DH/ER/KR/ACP	quinolidomicin congener
	1-1077	KS/AT_m_/DH/KR/ACP	
	1-1078	KS/AT_m_/KR/ACP-KS/AT_m_/KR/ACP-KS/AT_m_/KR/ACP	
	1-1080	KS/AT_m_/DH/ER/KR/ACP-KS/AT_mm_/DH/ER/KR/ACP-KS/AT_m_/DH/ER/KR/ACP-	
		KS/AT_m_/KR/ACP	
	1-1081	KS/AT_mm_/KR/ACP-KS/AT_m_/DH/KR/ACP-KS/AT_m_/DH/KR/ACP-	
		KS/AT_m_/DH/KR/ACP-KS/AT_m_/KR/ACP-KS/AT_m_/KR/ACP	
	1-1091	KS/AT_m_/KR/ACP-KS/AT_mm_/DH/KR/ACP	
	1-1092	KS/AT_m_/DH/KR/ACP-KS/AT_mm_/KR/ACP-KS/AT_m_/KR/ACP	
	1-1093	KS/AT_m_/KR/ACP-KS/AT_mm_/DH/ER/KR/ACP	
	1-1094	KS/AT_m_/KR/ACP- KS/AT_mm_/KR/ACP- KS/AT_mm_/KR/ACP	
	1-1095	KS/AT_m_/DH/KR/ACP-KS/AT_mm_/DH/KR/ACP	
	1-1096	KS/AT_mm_/KR/ACP	
	1-1097	KS/AT_mm_/KR/ACP-KS/AT_m_/KR/ACP	
	1-1098	KS/AT_m_/ACP-Te	
*t1pks-3*	4-330	KS/AT/KR/DH	sporolide
*t1pks-4* ^P^	14-64 ^P^	KS/AT…	unpredictable
	16-1 ^P^	…KR	
	16-2	KS/AT	
	16-3	ACP	
*t2pks-1*	4-99	KSα	aromatic polyketide
	4-100	KSβ (CLF)	
	4-101	ACP	
*t3pks-1* *	2-674	KS	alkyl-O-dihydrogeranyl-methoxyhydroquinone
*nrps-1*	1-336	C/A/T-C/A_thr_/T/E	pentapeptide (x-thr-phe-ser-ile)
	1-337	C/A_phe_/T/E-C/A_ser_/T	
	1-339	C/A_ile_/T	
*nrps-2*	4-510	C-C	tripeptide (x-gly-x)
	4-511	C/A/T-Te	
	4-512	A	
	4-513	C/A_gly_/T	
	4-514	T	
*nrps-3*	6-252	C/A_cys_/T-Te	tetrapeptide (x-x-cys-cys)
	6-258	A-C/A_cys_/T	
	6-265	C	
	6-266	A	
	6-270	A/T	
*nrps-4* ^P^	8-247	A/T-C	unpredictable
	8-248	A/T	
*nrps-5*	12-31	C/A_ser_/T-C/A_pro_/T-Te	ser-pro
*pks/nrps-1*	4-217 4-220 4-223 4-227 4-228 4-229 4-247	CoL/ACP-KS/AT_m_/ACP-C/A/T-C A_ala_/T-C A_glu_/T-C/T A_thr_/T T-C A_ser_/T-C/T-Te A/T	heptapeptide with polyketide moieties (st-pk-x-ala-glu-y-thr-ser-y)
*pks/nrps-2* *	6-50 6-51 6-52 6-53 6-54 6-55	C/A/T KS ACP C/A_val_/T KS/AT_m_/ACP A_ser_	tripeptide with polyketide moiety (ser-x-val-pk)
*pks/nrps-3* *	6-307 6-310 6-311 6-313 6-314	A/T-KS/DH A/T-C/T KS/AT_m_/KR/DH/ACP C/A_asn_/T C/A_ser_/T-Te	pentapeptide with polyketide moiety (x-pk-x-y-pk-asn-ser)
*pks/nrps-4*	8-41 8-40 8-39 8-37	A_gly_/T-KS/ACP-KS/AT_m_ DH/KR/ACP-KS/ACP-KS/KR/ACP KS/DH/ACP-KS/AT_m_ DH/KR/ACP-AmT	hexaketide with gly

^P^, not completely sequenced; *, conserved between strains TP-A0316 and TP-A0468; A, adenylation; ACP, acyl carrier protein; AmT, aminotransferase; AT, acyltransferase; AT_m_, AT for malonyl-CoA, AT_mm_, AT for methyl malonyl-CoA; C, condensation; CLF, chain length factor; CoL, CoA ligase; DH, dehydratase; dhb, dihydroxybenzoate; E, epimerization; ER, enoyl reductase; KR, ketoreductase; KS, ketosynthase; MT, methyltransferase; *nrps*, NRPS gene; pk, polyketide; *pks/nrps*, hybrid PKS/NRPS gene; st, starter molecule; T, thiolation; TD, termination; Te, thioesterase, *t1pks*, type-I PKS gene; *t2pks*, type-II PKS gene; *t3pks*, type-III PKS gene; x, unidentified amino acid residue; X, unknown domain, y, unknown unit by lack of A domain in the module. Amino acids incorporated by A domains are indicated by 3-letter abbreviations in subscript just after A. Most similar, known clusters (similarity in KnownClusterBlast) of *t1pks-1*, *t1pks-3* and *t3pks-1* are BGCs of tetrocarcin A (91%), sporolide (23%) and alkyl-O-dihydrogeranyl-methoxyhydroquinones (57%), respectively, by antiSMASH.

**Table 3:** Domain organizations of PKSs in quinolidomycin-BGC and *t1pks-2*.

M *	Quinolidomicin (*qnm*)	*t1pks-2*	
	in *M. chalcea* AK-AN57	in *Micromonospora* sp. TP-A0316	in *M. okii* TP-A0468^T^
	(qnmA1)	(1-1073)	(8-118)
L	CoL/ACP	CoL/KR/ACP	CoL/ACP
1	KS/ATm/DH/KR/ACP	KS/ATm/DH/KR/ACP	KS/ATm/DH/KR/ACP
2	KS/ATmm/DH/ER/KR/ACP	KS/ATmm/DH/ER/KR/ACP	KS/ATmm/DH/ER/KR/ACP
	(*qnmA2*)	(1-1077)	(8-122)
3	KS/AT_m_/DH/KR/ACP	KS/AT_m_/DH/KR/ACP	KS/AT_m_/DH/KR/ACP
	(*qnmA3*)	(1-1078)	(8-123)
4 5 6 7	**KS/AT_m_/DH/KR/ACP** KS/AT_m_/KR/ACP KS/AT_m_/KR/ACP KS/AT_m_/KR/ACP	- KS/AT_m_/KR/ACP KS/AT_m_/KR/ACP KS/AT_m_/KR/ACP	- KS/AT_m_/KR/ACP KS/AT_m_/KR/ACP KS/AT_m_/KR/ACP
	(*qnmA4*)	(1-1080)	(8-135)
8 9 10 11	KS/AT_m_/DH/ER/KR/ACP KS/AT/DH/ER/KR/ACP KS/AT_m_/DH/KR/ACP KS/AT_m_/KR/ACP	KS/AT_m_/DH/ER/KR/ACP KS/AT_mm_/DH/ER/KR/ACP KS/AT_m_/DH/ER/KR/ACP KS/AT_m_/KR/ACP	KS/AT_m_/DH/ER/KR/ACP KS/AT/DH/ER/KR/ACP KS/AT_m_/DH/ER/KR/ACP KS/AT_m_/KR/ACP
	(*qnmA5*)	(1-1081)	(8-136)
12	KS/AT_m_/KR/ACP	KS/AT_mm_/KR/ACP	KS/AT_mm_/KR/ACP
			(8-137)
13	KS/AT_m_/DH/KR/ACP	KS/AT_m_/DH/KR/ACP	KS/AT_m_/DH/KR/ACP
14	KS/AT_m_/DH/KR/ACP	KS/AT_m_/DH/KR/ACP	KS/AT_m_/DH/KR/ACP
15	KS/AT_m_/DH/KR/ACP	KS/AT_m_/DH/KR/ACP	KS/AT_m_/DH/KR/ACP
16	KS/AT_m_/KR/ACP	KS/AT_m_/KR/ACP	KS/AT_m_/KR/ACP
17	KS/AT_m_/KR/ACP	KS/AT_m_/KR/ACP	KS/AT_m_/KR/ACP
	(*qnmA6*)	(1-1091)	(8-146)
18 19	KS/AT_m_/KR/ACP *KS/DH/KR/ACP*	KS/AT_m_/KR/ACP KS/AT_mm_/DH/KR/ACP	KS/AT_m_/KR/ACP KS/AT_mm_/DH/KR/ACP
	(*qnmA7*)	(1-1092)	(8-147)
20 21 22	KS/AT_m_/DH/KR/ACP KS/AT_mm_/KR/ACP KS/AT_m_/KR/ACP	KS/AT_m_/DH/KR/ACP KS/AT_mm_/KR/ACP KS/AT_m_/KR/ACP	KS/AT_m_/DH/KR/ACP *KS/KR/ACP* KS/AT_m_/KR/ACP
	(*qnmA8*)	(1-1093)	(8-148)
23 24	KS/AT_m_/KR/ACP KS/AT_mm_/DH/ER/KR/ACP	KS/AT_m_/KR/ACP KS/AT_mm_/DH/ER/KR/ACP	KS/AT/KR/ACP KS/AT_mm_/DH/ER/KR/ACP
	(*qnmA9*)	(1-1094)	(8-149)
25	KS/AT_m_/KR/ACP	KS/AT_m_/KR/ACP	*KS/AT_m_*
			(8-150)
26 27	KS/AT_mm_/KR/ACP KS/AT_mm_/KR/ACP	KS/AT_mm_/KR/ACP KS/AT_mm_/KR/ACP	KS/AT_mm_/KR/ACP KS/AT_mm_/KR/ACP
	(*qnmA10*)	(1-1095)	(8-151)
28 29	KS/AT_m_/DH/KR/ACP KS/AT_mm_/DH/KR/ACP	KS/AT_m_/DH/KR/ACP KS/AT_mm_/DH/KR/ACP	KS/AT_m_/DH/KR/ACP KS/AT_mm_/DH/KR/ACP
	(*qnmA11*)	(1-1096)	(8-152)
30	KS/AT_mm_/KR/ACP	KS/AT_mm_/KR/ACP	KS/AT_mm_/KR/ACP
	(*qnmA12*)	(1-1097)	(8-153)
31 32	KS/AT_mm_/KR/ACP KS/AT_m_/KR/ACP	KS/AT_mm_/KR/ACP KS/AT_m_/KR/ACP	KS/AT_mm_/KR/ACP KS/AT_m_/KR/ACP
	(*qnmA13*)	(1-1098)	(8-154)
33	KS/AT_m_/**KR**/ACP/Te	KS/AT_m_/ACP/Te	KS/AT_m_/ACP/Te

* M, module; ACP, acyl carrier protein; AT, acyltransferase; AT_m_ for malonyl-CoA, AT_mm_, AT for methyl malonyl-CoA; CoL, CoA ligase; DH, dehydratase; ER, enoyl reductase; KR, ketoreductase; KS, ketosynthase; L, loading; Te, thioesterase; -, absent. Boldfaced and underlined domains are not observed in the others. Genes names and ORF no. are shown in parentheses. The domain organizations were surveyed by antiSMASH. Domain organizations in italicized modules may be doubtful because antiSMASH surrounded them by dashed lines.

**Table 4:** PKS and NRPS gene clusters in the genomes of *M. okii* TP-A0468^T^.

Gene Cluster	ORF	Domain Organization	Predicted Product
*t1pks-2* *	8-118	CoL/ACP-KS/AT_m_/DH/KR/ACP-KS/AT_mm_/DH/ER/KR/ACP	quinolidomicin congener
	8-122	KS/AT_m_/DH/KR/ACP	
	8-123	KS/AT_m_/KR/ACP-KS/AT_m_/KR/ACP-KS/AT_m_/KR/ACP	
	8-135	KS/AT_m_/DH/ER/KR/ACP-KS/AT/DH/ER/KR/ACP-	
		KS/AT_m_/DH/ER/KR/ACP-KS/AT_m_/KR/ACP	
	8-136	KS/AT_mm_/KR/ACP	
	8-137	KS/AT_m_/DH/KR/ACP-KS/AT_m_/DH/KR/ACP-KS/AT_m_/DH/KR/ACP-	
		KS/AT_m_/KR/ACP-KS/AT_m_/KR/ACP	
	8-146 8-147 8-148 8-149 8-150 8-151 8-152 8-153 8-154	KS/AT_m_/KR/ACP-KS/AT_mm_/DH/KR/ACP KS/AT_m_/DH/KR/ACP-KS/KR/ACP-KS/AT_m_/KR/ACP KS/AT/KR/ACP-KS/AT_mm_/DH/ER/KR/ACP KS/AT_m_ KS/AT_mm_/KR/ACP-KS/AT_mm_/KR/ACP KS/AT_m_/DH/KR/ACP-KS/AT_mm_/DH/KR/ACP KS/AT_mm_/KR/ACP KS/AT_mm_/KR/ACP-KS/AT_m_/KR/ACP KS/AT_m_/ACP-Te	
*t1pks-5*	17-167	CoL/ACP-KS/AT_mm_/DH/KR/ACP-KS/AT_mm_/ACP-KS/AT_mm_/KR/ACP	16-demethylrifamycins
		KS/AT_mm_/DH/KR/ACP-KS/AT_mm_/DH/KR/ACP-KS/AT_mm_/DH/KR/ACP	
	17-166	KS/AT_mm_/DH/KR/ACP	
		KS/AT_mm_/DH/KR/ACP	
	17-165	KS/AT_m_/DH/KR/ACP-KS/AT_m_/DH/KR/ACP	
	17-164		
	17-163		
*t2pks-2*	8-66 8-67 8-68	KSα KSβ (CLF) ACP	kosinostatin
*t2pks-3*	15-39 15-40 15-41	KSα KSβ (CLF) ACP	aromatic polyketide
*t3pks-1* *	13-182	KS	alkyl-O-dihydrogeranyl-methoxyhydroquinone
*t3pks-2*	9-577	KS	polyketide with guanidinotide moiety
*nrps-6* ^P^	8-1 ^P^ 8-2 8-14	…T C/A_cys_/MT/T A_dhb_	pyochelin
*nrps-7*	9-387	A_glu_/T-TD	glu with β-lactone
*nrps-8*	16-60 16-59 16-58	T A_val_ C/A_pro_/T-TD	dipeptide (val-pro)
*nrps-9*	19-118 19-110	A/T C/A/T	dipeptide (x-x)
*nrps-10*	20-72 20-83	A A/T/E	dipeptide (x-x)
*pks/nrps-2* *	24-73 24-74 24-75 24-76 24-77 24-78	C/A/T KS ACP C/A_val_/T KS/AT_m_/ACP A_ser_	tripeptide with polyketide moiety (ser-x-val-pk)
*pks/nrps-3* *	17-59 17-56 17-55 17-52 17-51	A/T-KS/DH A/T-C/T KS/AT_m_/KR/DH/ACP C/A_asn_/T C/A_ser_/T-Te	pentapeptide with polyketide moiety (x-pk-x-y-pk-asn-ser)
*pks/nrps-5*	21-43 21-44 21-45 21-46 21-47 21-48 21-50 21-51 21-53 21-62	C/A_asn_/T-C/A/T C/A_ser_/T KS/AT_m_/MT/KR/ACP C/A/T CoL/T-C/A_ser_/T-C T-C C/A_b-ala_/T-C/A_cys_/T-C A/T C A/T	methyltallysomycin
*pks/nrps-6*	26-56	A/T-KS/AT_m_/ACP-C/A/T-C	dipeptide with polyketide moiety (x-pk-x)

Footnotes are the same as those of Table 2. ^P^, not completely sequenced; *, conserved between strains TP-A0316 and TP-A0468. Most similar known cluster (similarity in KnownClusterBlast) of *t1pks-5, t2pks-2, t3pks-1* and *pks/nrps-5* are biosynthetic gene clusters of rifamycin (64%), kosinostatin (100%), alkyl-*O*-dihydrogeranyl-methoxyhydroquinones (57%) and tallysomycin (37%), respectively, by antiSMASH.

## Data Availability

The whole genome shotgun project of *Micromonospora* sp. TP-A0316 and *Micromonospora* sp. TP-A0468 have been deposited in DDBJ under the accession numbers BBOL00000000 and BBZF00000000, respectively. BioProject accession numbers are PRJDB3173 and PRJDB4094. BioSample accession numbers are SAMD00020840 and SAMD00035862.

## References

[1] Berdy J. (2005). Bioactive microbial metabolites. J. Antibiot..

[2] Qi S., Gui M., Li H., Yu C., Li H., Zeng Z., Sun P. (2020). Secondary metabolites from marine *Micromonospora*: Chemistry and bioactivities. Chem. Biodivers..

[3] Furumai T., Takagi K., Igarashi Y., Saito N., Oki T. (2000). Arisostatins A and B, new members of tetrocarcin class of antibiotics from *Micromonospora* sp. TP-A0316. I. Taxonomy, fermentation, isolation and biological properties. J. Antibiot..

[4] Furumai T., Igarashi Y., Higuchi H., Saito N., Oki T. (2002). Kosinostatin, a quinocycline antibiotic with antitumor activity from *Micromonospora* sp. TP-A0468. J. Antibiot..

[5] Fang J., Zhang Y., Huang L., Jia X., Zhang Q., Zhang X., Tang G., Liu W. (2008). Cloning and characterization of the tetrocarcin A gene cluster from *Micromonospora chalcea* NRRL 11289 reveals a highly conserved strategy for tetronate biosynthesis in spirotetronate antibiotics. J. Bacteriol..

[6] Ma H., Zhou Q., Tang Y., Zhang Z., Chen Y., He H., Pan H., Tang M., Gao J., Zhao S. (2013). Unconventional origin and hybrid system for construction of pyrrolopyrrole moiety in kosinostatin biosynthesis. Chem. Biol..

[7] Schwarzer D., Marahiel M.A. (2001). Multimodular biocatalysts for natural product assembly. Naturwissenschaften.

[8] Meurer G., Gerlitz M., Wendt-Pienkowski E., Vining L.C., Rohr J., Hutchinson C.R. (1997). Iterative type II polyketide synthases, cyclases and ketoreductases exhibit context-dependent behavior in the biosynthesis of linear and angular decapolyketides. Chem. Biol..

[9] Nett M., Ikeda H., Moore B.S. (2009). Genomic basis for natural product biosynthetic diversity in the actinomycetes. Nat. Prod. Rep..

[10] Komaki H., Oguchi A., Tamura T., Hamada M., Ichikawa N. (2020). Diversity of nonribosomal peptide synthetase and polyketide synthase gene clusters in the genus *Acrocarpospora*. J. Gen. Appl. Microbiol..

[11] Komaki H., Tamura T. (2020). Polyketide synthase and nonribosomal peptide synthetase gene clusters in type strains of the genus *Phytohabitans*. Life.

[12] Komaki H., Tamura T., Ichikawa N., Oguchi A., Hamada M., Suzuki K., Fujita N. (2015). Genome-based analysis of type-I polyketide synthase and nonribosomal peptide synthetase gene clusters in a novel strain taxonomically close to the genus *Salinispora*. J. Antibiot..

[13] Kasai H., Tamura T., Harayama S. (2000). Intrageneric relationships among *Micromonospora* species deduced from *gyrB*-based phylogeny and DNA relatedness. Int. J. Syst. Evol. Microbiol..

[14] Hatano K., Nishii T., Kasai H. (2003). Taxonomic re-evaluation of whorl-forming *Streptomyces* (formerly *Streptoverticillium*) species by using phenotypes, DNA-DNA hybridization and sequences of *gyrB*, and proposal of *Streptomyces luteireticuli* (*ex* Katoh and Arai 1957) corrig., sp. nov., nom. rev. Int. J. Syst. Evol. Microbiol..

[15] Fang B., Liu C., Guan X., Song J., Zhao J., Liu H., Li C., Ning W., Wang X., Xiang W. (2015). Two new species of the genus *Micromonospora*: *Micromonospora palomenae* sp. nov. and *Micromonospora harpali* sp. nov. isolated from the insects. Antonie Van Leeuwenhoek.

[16] Kittiwongwattana C., Thanaboripat D., Laosinwattana C., Koohakan P., Parinthawong N., Thawai C. (2015). *Micromonospora oryzae* sp. nov., isolated from roots of upland rice. Int. J. Syst. Evol. Microbiol..

[17] Xie Q., Ren J., Li L., Li Y., Deng Z., Hong K. (2016). *Micromonospora mangrovi* sp. nov., isolated from mangrove soil. Antonie Van Leeuwenhoek.

[18] Hashimoto T., Hashimoto J., Kozone I., Amagai K., Kawahara T., Takahashi S., Ikeda H., Shin-ya K. (2018). Biosynthesis of quinolidomicin, the largest known macrolide of terrestrial origin: Identification and heterologous expression of a biosynthetic gene cluster over 200 kb. Org. Lett..

[19] McGlinchey R.P., Nett M., Moore B.S. (2008). Unraveling the biosynthesis of the sporolide cyclohexenone building block. J. Am. Chem. Soc..

[20] Awakawa T., Fujita N., Hayakawa M., Ohnishi Y., Horinouchi S. (2011). Characterization of the biosynthesis gene cluster for alkyl-*O*-dihydrogeranyl-methoxyhydroquinones in *Actinoplanes missouriensis*. ChemBioChem.

[21] Zhou Q., Luo G., Zhang H., Tang G. (2019). Discovery of 16-demethylrifamycins by removing the predominant polyketide biosynthesis pathway in *Micromonospora* sp. strain TP-A0468. Appl. Environ. Microbiol..

[22] Tao M., Wang L., Wendt-Pienkowski E., George N.P., Galm U., Zhang G., Coughlin J.M., Shen B. (2007). The tallysomycin biosynthetic gene cluster from *Streptoalloteichus hindustanus* E465-94 ATCC 31158 unveiling new insights into the biosynthesis of the bleomycin family of antitumor antibiotics. Mol. Biosyst..

[23] Braesel J., Crnkovic C.M., Kunstman K.J., Green S.J., Maienschein-Cline M., Orjala J., Murphy B.T., Eustaquio A.S. (2018). Complete genome of *Micromonospora* sp. strain B006 reveals biosynthetic potential of a Lake Michigan actinomycete. J. Nat. Prod..

[24] Komaki H., Sakurai K., Hosoyama A., Kimura A., Igarashi Y., Tamura T. (2018). Diversity of nonribosomal peptide synthetase and polyketide synthase gene clusters among taxonomically close *Streptomyces* strains. Sci. Rep..

[25] Wayne L.G., Brenner D.J., Colwell R.R., Grimont P.A.D., Kandler O., Krichevsky M.I., Moore L.H., Moore W.E.C., Murray R.G.E., Stackebrandt E. (1987). Report of the ad hoc committee on reconciliation of approaches to bacterial systematics. Int. J. Syst. Bacteriol..

[26] Weinstein M.J., Luedemann G.M., Oden E.M., Wagman G.H., Rosselet J.P., Marquez J.A., Coniglio C.T., Charney W., Herzog H.L., Black J. (1963). Gentamicin, a new antibiotic complex from *Micromonospora*. J. Med. Chem..

[27] Testa R.T., Wagman G.H., Daniels P.J., Weinstein M.J. (1974). Mutamicins; biosynthetically created new sisomicin analogues. J. Antibiot..

[28] Weinstein M.J., Marquez J.A., Testa R.T., Wagman G.H., Oden E.M., Waitz J.A. (1970). Antibiotic 6640, a new *Micromonospora*-produced aminoglycoside antibiotic. J. Antibiot..

[29] Zhang F., Zhao M., Braun D.R., Ericksen S.S., Piotrowski J.S., Nelson J., Peng J., Ananiev G.E., Chanana S., Barns K. (2020). A marine microbiome antifungal targets urgent-threat drug-resistant fungi. Science.

[30] Hayakawa Y., Matsuoka M., Shin-ya K., Seto H. (1993). Quinolidomicins A1, A2 and B1, novel 60-membered macrolide antibiotics. I. Taxonomy, fermentation, isolation, physico-chemical properties and biological activity. J. Antibiot..

[31] Hayakawa Y., Shin-ya K., Furihata K., Seto H. (1993). Quinolidomicins A1, A2 and B1, novel 60-membered macrolide antibiotics. II. Structure elucidation. J. Antibiot..

[32] Komaki H., Ichikawa N., Oguchi A., Hamada M., Harunari E., Kodani S., Fujita N., Igarashi Y. (2016). Draft genome sequence of *Streptomyces* sp. TP-A0867, an alchivemycin producer. Stand. Genom. Sci..

[33] Yoon S., Ha S., Kwon S., Lim J., Kim Y., Seo H., Chun J. (2017). Introducing EzBioCloud: A taxonomically united database of 16S rRNA gene sequences and whole-genome assemblies. Int. J. Syst. Evol. Microbiol..

[34] Alanjary M., Steinke K., Ziemert N. (2019). AutoMLST: An automated web server for generating multi-locus species trees highlighting natural product potential. Nucleic Acids Res..

[35] Komaki H., Ichikawa N., Hosoyama A., Fujita N., Igarashi Y. (2015). Draft genome sequence of marine-derived *Streptomyces* sp. TP-A0598, a producer of anti-MRSA antibiotic lydicamycins. Stand. Genom. Sci..

[36] Komaki H., Ishikawa A., Ichikawa N., Hosoyama A., Hamada M., Harunari E., Nihira T., Panbangred W., Igarashi Y. (2016). Draft genome sequence of *Streptomyces* sp. MWW064 for elucidating the rakicidin biosynthetic pathway. Stand. Genom. Sci..

[37] Komaki H., Sakurai K., Hosoyama A., Kimura A., Trujilo M.E., Igarashi Y., Tamura T. (2020). Diversity of PKS and NRPS gene clusters between *Streptomyces abyssomicinicus* sp. nov. and its taxonomic neighbor. J. Antibiot..

[38] Blin K., Shaw S., Steinke K., Villebro R., Ziemert N., Lee S.Y., Medema M.H., Weber T. (2019). antiSMASH 5.0: Updates to the secondary metabolite genome mining pipeline. Nucleic Acids Res..

[39] Meier-Kolthoff J.P., Auch A.F., Klenk H.P., Göker M. (2013). Genome sequence-based species delimitation with confidence intervals and improved distance functions. BMC Bioinform..

